# Ready for Discharge? A Survey of Discharge Transition-of-Care Education and Evaluation in Emergency Medicine Residency Programs

**DOI:** 10.5811/westjem.2015.9.27298

**Published:** 2015-11-12

**Authors:** Fiona E. Gallahue, Amy E. Betz, Jeffrey Druck, Jonathan S. Jones, Boyd Burns, Gene Hern

**Affiliations:** * The University of Washington, Division of Emergency Medicine, Seattle, Washington; † The University of Colorado, Department of Emergency Medicine, Aurora, Colorado; ‡ The University of Mississippi Medical Center, Department of Emergency Medicine, Jackson, Mississippi; § The University of Oklahoma School of Community Medicine, Tulsa, Oklahoma; ¶ Alameda Hospital System-Highland Hospital, Department of Emergency Medicine, Oakland, California

## Abstract

This study aimed to assess current education and practices of emergency medicine (EM) residents as perceived by EM program directors to determine if there are deficits in resident discharge handoff training. This survey study was guided by the Kern model for medical curriculum development. A six-member Council of EM Residency Directors (CORD) Transitions of Care task force of EM physicians performed these steps and constructed a survey. The survey was distributed to program residency directors via the CORD listserve and/or direct contact. There were 119 responses to the survey, which were collected using an online survey tool. Over 71% of the 167 American College of Graduate Medical Education (ACGME) accredited EM residency programs were represented. Of those responding, 42.9% of programs reported formal training regarding discharges during initial orientation and 5.9% reported structured curriculum outside of orientation. A majority (73.9%) of programs reported that EM residents were not routinely evaluated on their discharge proficiency. Despite the ACGME requirements requiring formal handoff curriculum and evaluation, many programs do not provide formal curriculum on the discharge transition of care or evaluate EM residents on their discharge proficiency.

Millions of patients are seen in the emergency department (ED) with approximately 86% rate of discharge.^1^ The discharge transition of care is the most commonly performed handoff in the ED and yet, most studies have focused on the handoffs between providers. Discharge is the handoff from provider responsibility to patient responsibility for care. This is a complex process representing a time of significant vulnerability for patients. Safe and effective transfer of responsibility for a patient’s medical care relies on effective provider communication with patient comprehension of discharge instructions. Studies have demonstrated that patients discharged from the ED have significant gaps in their understanding of this information.^2^,^3^ There is some literature to suggest that the quality of verbal communication at discharge by emergency providers is incomplete and leaves little room for patients to ask questions.^4^ There is evidence to suggest that ineffective communication between providers and patients is a source of error in the discharge period.^5^,^6^ It has been demonstrated that residents overestimate the effectiveness of their communication.^7^ Additionally, residents may not recognize patient factors that place patients at high risk for readmission.^8^

Evidence on how to ensure ideal handoffs has been limited, but multiple sources have identified process standardization as an opportunity for quality improvement.^9^,^10^ Accordingly, standardization of handoffs was made a National Patient Safety Goal by the Joint Commission in 2006.^11^ The American College of Graduate Medical Education (ACGME) has identified education and evaluation of care transitions as an educational mandate in training programs. The ACGME states “formal educational activities that create a shared mental model with regard to care transitions are necessary” and that “evaluation through direct observation of residents/fellows by faculty members is required to ensure residents’/fellows’ abilities to perform standardized, effective, efficient handoffs.”^12^

Although there are clear mandates to ensure that handoffs are standardized, and formal handoff curriculum and evaluation are provided to emergency medicine (EM) residents, there is no information available to identify the current practices of EM training programs on the discharge transition of care.

The objectives in this study were to (1) assess the current scope of discharge training among EM residency programs by surveying their residency leadership, (2) assess current educational and evaluation practices related to discharge training, and (3) identify whether additional training is necessary based on the current practices and perceived competencies.

## METHODS

This survey study was guided by the six-step Kern model for medical curriculum development.^13^ A similar study by the CORD Transitions of Care taskforce membership was performed on general handoff training, which provided a foundation for this study.^14^ The first two stages–problem identification and creation of a targeted needs assessment–were the goals of this study. The latter four stages of the Kern model include determining the goals and objectives of the curriculum, developing educational strategies for teaching the curriculum, implementing the curriculum, and receiving feedback on and evaluating the curriculum. The application of this model provides the opportunity to eventually develop a discharge handoff curriculum for EM residents. Establishing validity evidence was an important consideration throughout the process; validity evidence comes in the form of content, response process, internal structure, relationship to other variables, and consequences.^15^ We conducted a review of discharge literature to survey common practices and sources of error and to discover EM discharge education techniques. One identified problem was the lack of formal emergency discharge education available or required during residency training. The survey went through a thorough development process using the expertise of those involved in its development to contribute to the validity of its content.^15^ This process was an iterative approach by the authors who are on the Council of Residency Directors in EM (CORD) Transitions of Care Committee (FG, JD, JJ, BB, HGH) or have served as a director of Quality Assurance and Improvement (AB). The initial survey was developed by two authors (HGH, JD) based on focus group comments and suggestions from membership during a CORD Transitions of Care Committee meeting (approximately 20 faculty in attendance). We sent the draft survey to the authorship team via SurveyMonkey® (an online survey development cloud-based service) to complete and suggest further content changes. This was repeated twice more until the team felt that the final questionnaire best addressed the areas where knowledge content gaps were identified by the focus group. The survey focused on elucidating current practices of discharge training and clinical practices within the clinical environments, including current education offered, perceptions of best educational practices, methods of resident evaluation and perceived competence of residents. Multiple-choice questions were the primary vehicle for the response process.

The institutional review board at Alameda County Medical Center (Highland Hospital, Oakland, CA) granted exempt approval for this study. Members of CORD were invited to complete the survey electronically. The CORD e-mail listserve is exclusive to educators in EM residency programs and includes associate, assistant and primary residency program directors from the 167 ACGME-accredited EM residency programs. Program leaders were recruited via the CORD listserve from March to April 2014. The survey was opened for six weeks in which 87 identified programs responded. Duplicate responses were reviewed and clarified with program directors directly in April 2015 (22 programs). Two programs requested their duplicate responses be deleted and completed new surveys to accurately represent their current practice. All other programs selected their most accurate survey responses. Direct emails were then sent to program directors with links to the survey in April 2015; 32 additional programs completed the survey and the survey was closed on April 23, 2015. Only programs that identified their program name were included in the study to ensure that all surveyed programs represented ACGME-accredited programs and to avoid potential duplicative responses.

## RESULTS

A total of 119 programs were surveyed, making the overall response rate 71.2% of the 167 currently accredited ACGME EM residency training programs. A majority of programs indicated that residents are given informal education regarding discharge training by senior residents and faculty (87.4%); just under half provide formal curriculum at orientation (42.9%) and/or outside of orientation (5.9%). A small percentage of programs offer no training (6.7%) (Table 1). Over half of programs felt that optimal discharge training would be formal curriculum offered at orientation (76, 63.9%) and/or outside of orientation (63, 52.9%).

Most residency programs reported using a structured discharge system in the ED (100, 84%%), while a small minority report using none (13, 10.9%) or being unsure if this is provided in their main ED (4, 7.6%). For those with a structured process of discharge most reported that this is being performed most of the time (35, 35%) or always (56, 56%). A majority of programs reported providing structured written modifiable written discharge instructions (108, 90.8%) and bidirectional conversations with patients (91, 76.5%) (Table 2). A majority of training programs reported that their current process is safe and effective or extremely safe and effective (78, 66%) but a significant number reported their process to be somewhat safe and effective (41, 34%). Most programs do not formally evaluate their residents for discharge proficiency (88, 73.9%). Of those that reported resident evaluation of discharge proficiency is routinely performed (more than one response was acceptable), 25 programs (21%) report the evaluation is completed as part of required direct observations or other activities, nine (7.6%) reported it is completed through written feedback/evaluation of performance on ED rotations, and two programs (1.7%) reported a formal assessment of discharge proficiency is completed on junior residents as part of a checklist or similarly structured evaluation.

All programs reported a variety of tools to assist in the discharge process with only 34, or 43%, of the respondents being satisfied or extremely satisfied with these tools (Table 3). Over two-thirds of programs reported that key elements of discharge conversations, such as diagnosis, education, prescriptions, follow up, return precautions or assessment of understanding, are documented in the physician note (80, 67.2%). This information is not routinely documented in 31 programs (26.1%), and eight respondents were not sure if this is included in their documentation (6.7%).

Over three-quarters (90, 75.6%) of program leadership reported that junior level residents (equal to or less than eight months in the ED) are “somewhat competent” in their discharge competency. Almost a quarter felt that their junior residents were “competent” (28, 23.5%). One program (0.8%) reported their junior residents were extremely competent (1, 0.8%). For senior level residents, described as residents with over eight months experience, their program leadership identified them as competent (83, 68.7%) or extremely competent (26, 21.8%). A minority of programs reported their senior level residents as “somewhat competent” in their discharge skills (10, 8.4%). None of the respondents reported their junior or senior level residents to be incompetent in their discharge abilities.

## DISCUSSION

These results provide insight into the discharge educational practices and clinical training experience surrounding discharge of EM residents as reported by their program leadership. Standardized formal training and evaluation is not the current norm at most programs. Most formal training provided to EM residents is at their orientation, and few programs offer formal educational opportunities beyond the first few weeks of training. Since a majority of program leaders indicated that ideal educational practices would include formal training at orientation and/or workshops or classes outside of orientation, programs may value structured education of discharge competencies but may be constrained by other limitations such as faculty time, didactic scheduling or curriculum availability. The same gap was seen between current training and ideal training for evaluation processes. While most programs do not perform formal evaluations on their residents’ discharge competency, a majority of programs identify that this would be an ideal practice. This implies that EM program leadership values formal evaluation of their residents’ discharge competency but they may be constrained by limitations such as a recognized evaluation tool and/or faculty time. These data also suggest that while program leadership values discharge competency training and evaluations, this education may not be valued as a high priority since most programs perceive their senior level residents to be competent. Although it is difficult to fully endorse competency without a standardized evaluation process, there is support that informal evaluation may be valid in identifying residents’ clinical competencies.^16^

## LIMITATIONS

The fact that this study relies on perceptions of program leadership is a major limitation as there is no gold standard to formally measure discharge competency even for programs providing formal evaluation of their residents’ discharge competency. While most program leadership feel that their current process of discharge is “safe and effective,” this perception may be limited. Given that each respondent based their program results on his or her perceptions of the discharge education and performance of residents within the clinical environment, construct underrepresentation and irrelevant variance represent threats to the validity of clinical performance ratings in this study.^17^ Program leadership could have responded based on too few or incomplete observations of residents’ clinical behavior or responded with low-reliability ratings. Survey, rater and recall bias could have affected these results.

Program leadership relies on general gestalt that their residents are competent in their discharge proficiency since most are not evaluating this competency. Program leadership may not identify any limitations in senior resident discharge competency because there may be larger, more systematic failures of discharge communication with patients and/or caregivers. In this scenario, resident performance of discharge may be at an acceptable level at the departmental level but departmental expectations of patient discharge competency may not be meeting the patient needs to create safe and effective discharges. Standardizing the process of handoffs between providers in the hospital environment has demonstrated improvement in the quality of communication and increased patient safety in the clinical arena.^9^ Current discharge literature suggests that there may be similar communication improvements to be made in EM around discharge.^3^–^5^ That over one-third of programs report their current discharge process is only “somewhat safe and effective” suggest that there may be quality gaps in the current institutionally acceptable patient discharge processes.

The conflation of the concept of “safety” and “effectiveness” may be another limitation of this survey. Programs were asked about safe and effective discharges in a single question. These two concepts may be inappropriately linked together and it may be that safety may exist without being effective and vice versa. This may represent a construct error in the survey design and affected results of this survey.

Lastly, a major limitation of this study was the gap of approximately one year between the surveys of the first cohort of surveyed programs (87) and the second (32). Although it is unlikely that most programs changed their educational practices dramatically within that time period, it is possible.

Further work should focus on the more structured assessment of resident discharge competency through direct observation and evaluation of resident performance to corroborate program leaders’ assessment of resident competence. These evaluation tools should then be validated and studied in a clinical setting with specific process measures and patient outcomes. Following Kern’s six-step model for curriculum development, the next step would be to create specific goals and objectives of the discharge curriculum and develop educational interventions aligned with these goals. Curricular tool suggestions that have been made to structure education and evaluation for provider-to-provider handoffs might be used directly or modified to educate residents in the discharge transitions of care.^18^

## CONCLUSION

The results of this targeted needs assessment indicate a lack of structured training and assessment of resident discharge competency despite current guidelines for formalized training in all handoffs. Although most programs reported senior residents are competent in discharge proficiency, the residents’ training is primarily informal which may lead to significant variability in resident experience and performance. Further research should be aimed at assessing proficiency of resident discharge performance through objective observation with validated evaluation tools. Structured training and assessment recommendations should follow from this research with increased attention to implementing a standard curricular model or toolbox, objective, valid evaluation methods, and identification and management of high-risk discharges.

## Figures and Tables

**Table 1:** Transition-of-care survey results representing 119 respondents. Q1: Types of discharge training offered to residents/students (more than one response acceptable). Q2: What training around discharge processes do you believe would be best to provide to residents/students (more than one response acceptable).

Answer options	Percent response (count): Q1	Percent response (count): Q2
No training	6.7% (8)	1.7% (2)
Specific formal training regarding discharges during initial orientation to the program	42.9% (51)	63.9% (76)
Structured workshops/classes to teach proper discharge processes during residency (not in orientation)	5.9% (7)	52.9% (63)
Instruction by attending/senior resident within the clinical environment	87.4% (104)	69.7% (83)
Distributed educational packets/guides	9.2% (11)	18.5% (22)
Formal evaluation of residents on competency in performing effective discharges	14.3% (17)	58% (69)
Informal evaluation of residents on competency in performing effective discharges	39.5% (47)	24.4% (29)
Other	N/A (3)*	N/A (1)**

* 3 responses: 1) Discuss during Morbidity & Mortality conference. 2) Formal evaluation of effective discharges is covered loosely in chart reviews, call backs are performed intern year. 3) Grand rounds presentation of ED Discharges.

** 1 response: 1) I don’t know.

**Table 2:** Results represent 119 respondents. Q: Which of the following is included in your standard discharge process? More than one response is acceptable.

Answer options	Percent response (count)
Provision of any prewritten non-modifiable instructions	30.3% (36)
Provision of any structured written instructions that allow for modification	90.8% (108)
Physician routinely has bidirectional conversation with patient regarding diagnosis, education, prescriptions, follow up and reasons to return to the ED	76.5% (91)
Teach back method (or similar) routinely employed to assess patient understanding of their diagnosis, education, prescriptions, follow up and reasons to return to the ED	10.9% (13)
Final discharge routinely completed by nursing	68.9% (82)
Final discharge routinely completed by physician	10.9% (13)
Final discharge routinely completed by either nursing or physician	22.7% (27)
Other	N/A (2*)

*ED,* emergency department

* 2 responses: 1) Provide follow up physician or clinic. 2) Nursing employs teach back with patients.

**Table 3:** Q: Do you use any of the following tools to assist in the discharge process? More than one response is acceptable.

Answer options	Percent response (count)
Automated reminders within the computer interface	42.9% (51)
Written template or other written aids (badge card checklist)	24.4% (29)
Mnemonics	1.7% (2)
Teach back or similar method	3.4% (4)
None	38.7% (46)
Other	2*

* 2 responses: 1) Nursing feedback when discharge performed improperly. 2) Pre-populated recommendations from nursing triage such as smoking cessation for smokers, blood pressure re-check for patients with high blood pressure at triage; all patients without a primary care provider are provided a printout of the free and low cost medical, dental and mental health resources in the community.
